# ﻿Three new species of *Fuscoporia* (Hymenochaetales, Basidiomycota) from southern India revealed by morphological and multigene phylogenetic analyses

**DOI:** 10.3897/mycokeys.125.168173

**Published:** 2025-11-18

**Authors:** Sugantha Gunaseelan, Kezhocuyi Kezo, Elangovan Arumugam, Samantha C. Karunarathna, Al-Bandari Fahad Al-Arjani, Abdallah M. Elgorban, Jaturong Kumla, Nakarin Suwannarach, Ekachai Chukeatirote, Malarvizhi Kaliyaperumal

**Affiliations:** 1 Centre for Advanced Studies in Botany, University of Madras, Guindy Campus, Chennai 600025, Tamil Nadu, India; 2 Center for Yunnan Plateau Biological Resources Protection and Utilization & Yunnan International Joint Laboratory of Fungal Sustainable Utilization in South and Southeast Asia, College of Biology and Food Engineering, Qujing Normal University, Qujing 655099, China; 3 Botany and Microbiology Department, College of Science, King Saud University, Riyadh 11451, Saudi Arabia; 4 Center of Excellence in Biotechnology Research (CEBR), DSR, King Saud University, Riyadh, Saudi Arabia; 5 Center of Excellence in Microbial Diversity and Sustainable Utilization, Chiang Mai University, Chiang Mai 50200, Thailand; 6 School of Science, Mae Fah Luang University, Chiang Rai, 57100, Thailand

**Keywords:** Hymenochaetaceae, multigene phylogeny, *Phellinus* sensu lato, taxonomy, wood-rotting fungi

## Abstract

*Fuscoporia* Murrill, a cosmopolitan genus of the Hymenochaetaceae, consists of parasitic and saprotrophic fungi characterized by resupinate to pileate, strictly dimitic hyphal systems, encrusted generative hyphae, the presence of hymenial setae, and hyaline, smooth, thin-walled basidiospores. Based on morpho-microtaxonomic examinations and phylogenetic analyses using a combined ITS, nrLSU, partial *rpb2*, and *tef1-α* dataset, three new species of *Fuscoporia*—*F. indica*, *F. sirumalaiensis*, and *F. terminalianae*—are described from the Eastern Ghats of Tamil Nadu, India. The newly described species form three distinct lineages within the *F. torulosa* complex. *Fuscoporia indica* is characterized by imbricate, convex, dimidiate basidiomes, a glabrous, azonate pilear surface, and ellipsoid basidiospores (3.5–4.5 × 2.5–2.9 μm). *Fuscoporia sirumalaiensis* has a smooth to glabrous, concentrically zonate pilear surface, a duplex context, and smaller basidiospores (3.1–3.6 × 2.1–2.6 μm). Finally, *F. terminalianae* is recognized by its effused-reflexed to imbricate basidiome, widely zonate and warted pilear surface, and ellipsoidal basidiospores (3.3–4.3 × 2.8–3 μm). This study provides comprehensive descriptions, morphological illustrations, and insights into the differences among these new species and their respective allied taxa, along with the results of phylogenetic analysis.

## ﻿Introduction

The genus *Fuscoporia* Murrill was erected with *F. ferruginosa* (Schrad.) Murrill as the type species (Murrill 1907). It is widely recognized as a forest pathogen that causes white rot in both coniferous and deciduous trees (Panconesi et al. 1994; Spirin et al. 2014; Luana et al. 2015). *Fuscoporia* is characterized by its annual to perennial, resupinate to pileate basidiomes, a dimitic hyphal system with encrusted generative hyphae, and the presence of hymenial setae in most species except for *F. shoreae*, *F. longisetulosa*, and *F. discipes*. Marginal setae may be present or absent, and the basidiospores are hyaline, thin-walled, smooth, and range in shape from subglobose to cylindrical (Dai 2010; Wu et al. 2022). *Fuscoporia* is distributed worldwide; more than 1,561 nucleotide sequences belonging to 63 known and 55 unspecified species are publicly available in GenBank (Sayers et al. 2025), and 130 taxa are registered in MycoBank (Robert et al. 2013) as of October 2025.

Phylogenetic studies of North American and European members of the Hymenochaetaceae based on nrLSU redefined *Phellinus* sensu lato into several monophyletic genera. These include *Fomitiporia* Murrill, *Fomitiporella* Murrill, *Fulvifomes* Murrill, *Fuscoporia* Murrill, *Phellinus* s. str., *Porodaedalea* Murrill, *Phellinidium* (Kotl.) Fiasson and Niemelä, *Phellopilus* Niemelä, T. Wagner and M. Fisch., *Aurificaria* D.A. Reid, *Onnia* P. Karst., and *Phylloporia* Murrill (Fiasson and Niemelä 1984; Fischer 1996; Niemelä et al. 2001; Wagner and Fischer 2001, 2002), all of which were later accepted as distinct taxa (Niemelä et al. 2001; Spirin et al. 2006; Groposo et al. 2007; Baltazar et al. 2009; Baltazar and Gibertoni 2010; Dai 2010; Raymundo et al. 2013; Chen and Yuan 2017; Chen et al. 2019; Chen and Dai 2019). Recent molecular analyses of *Fuscoporia* have revealed that within this taxon, there are several major groupings supported by substantial morphological and phylogenetic evidence. These are the *F. contigua* complex, the *F. ferra* complex, the *F. ferruginosa* complex, the *F. gilva* complex, the *F. torulosa* complex, and the *F. viticola* complex (Chen et al. 2022, 2024; Wu et al. 2022; Cho et al. 2023).

Chen et al. (2019, 2020, 2023a, b) proposed 18 new species—*F. acutimarginata*, *F. australasica*, *F. australiana*, *F. americana*, *F. bambusae*, *F. centroamericana*, *F. chinensis*, *F. costaricana*, *F. eucalypti*, *F. karsteniana*, *F. latispora*, *F. monticola*, *F. plumeriae*, *F. ramulicola*, *F. septiseta*, *F. sinica*, *F. shoreae*, and *F. subchrysea*—from Australia, China, Costa Rica, Mexico, Singapore, Thailand, and the United States. From 2022 to 2024, numerous new species—*F. dolichoseta*, *F. dollingeri*, *F. eucalypticola*, *F. gilvoides*, *F. hawaiiana*, *F. koreana*, *F. minutissima*, *F. naditirana*, *F. resupinate*, *F. reticulata*, *F. semicephala*, *F. sinuosa*, *F. submurina*, and *F. subtropica*—have been reported worldwide (Wu et al. 2022; Chen et al. 2023a, b; Cho et al. 2023; Chen et al. 2024; Crous et al. 2024). Recently, Chen et al. (2025) reported *Fuscoporia reflexoides*, a new species belonging to the *F. gilva* complex from China.

Despite significant advancements, several regions of the world remain under-sampled, including Oceania, parts of South America, tropical Africa, and Southeast Asian islands. For a more comprehensive understanding of *Fuscoporia*, widespread sampling from Neotropical, Paleotropical, tropical, and temperate countries of Asia is essential. Earlier, knowledge of the species diversity of hymenochaetoid fungi in India was confined to the northern regions, primarily based on morpho-taxonomic characteristics. Kumari et al. (2021) documented 14 species of *Phellinus* (*P. callimorphus*, *P. chryseus*, *P. contiguus*, *P. discipes*, *P. ferreus*, *P. ferruginosus*, *P. gilvus*, *P. orientalis*, *P. punctatiformis*, *P. rhabarbarinus*, *P. rufitinctus*, *P. senex*, *P. torulosus*, and *P. wahlbergii*), which have since been validated and transferred to *Fuscoporia* through phylogenetic analysis. From the southern region of India, Bakshi (1971) reported *P. discipes* (syn. *F. discipes*). Our previous study represented the first multigene approach in revealing *F. naditirana* from the Eastern Ghats of Tamil Nadu (Crous et al. 2024). In the present study, we report three novel species of *Fuscoporia* from the Eastern Ghats of Tamil Nadu and provide detailed descriptions, illustrations, and phylogenetic placement based on ITS+nrLSU+*rpb2*+*tef1-α* sequence data.

## ﻿Materials and methods

### ﻿Morphological analyses

The studied specimens were collected from various locations in the Eastern Ghats of Tamil Nadu, India. Morphological and microscopic characteristics were analyzed as described earlier (Gunaseelan et al. 2024), and the phenetic color codes follow the *Methuen Handbook* (Kornerup and Wanscher 1978). Freehand sections of dried basidiomes were mounted in water, 5% potassium hydroxide (KOH), cotton blue (CB), and Melzer’s reagent (IKI) to analyze micro-morphological features. Microscopic observations, measurements, and line drawings were performed in 5% KOH using a LABOMED CxL2 compound microscope. Photomicrographs were taken with a LABOMED OPTIC-CX BINO LED microscope at magnifications up to 1000×. Basidiospore measurements (minimum–mean–maximum) and Q values (length/width ratios) were recorded. Abbreviations: CB^−^ = acyanophilous, CB^+^ = cyanophilous, IKI^−^ = inamyloid, IKI^+^ = amyloid, Q = L/W ratio (basidium length excluding sterigmata), L = mean spore length (arithmetic mean of all spores), W = mean spore width (arithmetic mean of all spores), n = number of spores measured. Measurements were based on 50 basidiospores, 30 cystidioles, hymenial setae, basidioles, and basidia per specimen. The identified specimens were deposited at the Madras University Botany Laboratory (MUBL), Centre for Advanced Studies in Botany, University of Madras, Chennai 600025, Tamil Nadu, India.

### ﻿Genomic DNA extraction, PCR amplification, and sequencing

The nuclear ribosomal internal transcribed spacer (ITS) region was amplified using primers ITS1 and ITS4 (White et al. 1990). PCR amplification was carried out under the following conditions: initial denaturation at 95 °C for 3 minutes, followed by 32 cycles of 95 °C for 30 seconds, 52 °C for 30 seconds, and 68 °C for 1 minute, with a final extension at 68 °C for 3 minutes. The nuclear ribosomal large subunit (nrLSU) region was amplified using primers LR0R and LR7 (Vilgalys and Hester 1990). PCR amplification conditions for nrLSU were as follows: initial denaturation at 94 °C for 1 minute, followed by 34 cycles at 94 °C for 30 seconds, 45 °C for 30 seconds, and 72 °C for 1.5 minutes, and a final extension at 72 °C for 10 minutes. The RNA polymerase II gene (*rpb2*) was amplified with primers bRPB2-6F and bRPB2-7.1R (Matheny 2005), and PCR conditions were as follows: initial denaturation at 94 °C for 2 minutes; 35 cycles of 94 °C for 45 seconds, 53 °C for 90 seconds, and 72 °C for 90 seconds; and a final extension at 72 °C for 10 minutes. The translation elongation factor 1-alpha gene (*tef1-α*) was amplified with primer pair EF1-983F/EF1-1567R (Rehner and Buckley 2005). PCR amplification conditions for *tef1-α* were as follows: initial denaturation at 94 °C for 3 minutes, followed by 34 cycles of denaturation at 94 °C for 40 seconds, annealing at 54 °C for 45 seconds, extension at 72 °C for 1 minute, and a final extension at 72 °C for 10 minutes. Sequencing was outsourced to Eurofins Genomics India Pvt. Ltd., Karnataka, India.

### ﻿Phylogenetic analyses

For the phylogenetic analyses, concatenated sequences (ITS+nrLSU+*rpb2*+*tef1-α*) generated from this study and allied taxa retrieved from GenBank (Table 1), along with the outgroups *Coniferiporia weirii* (CFS 504) and *Phellinidium fragrans* (CBS 202.90), were aligned in MEGA X v10.0.2, followed by manual adjustments to enhance alignment accuracy (Kumar et al. 2018). To facilitate dataset partitioning, the most appropriate partitioning scheme and substitution models were determined using PartitionFinder v1.10 (Lanfear et al. 2012) under the AIC criterion with the “greedy” search option. The dataset was subdivided into 10 partitions: ITS1, 5.8S, ITS2, nrLSU, *rpb2* introns, *rpb2* exon, *tef1-α* introns, *tef1-α* 1^st^ codon, *tef1-α* 2^nd^ codon, and *tef1-α* 3^rd^ codon. The best-fit evolutionary models selected by MrModeltest v2.3 (Nylander 2004) were implemented for each partition: GTR+G (ITS1), GTR+I+G (5.8S), JC69 (ITS2), HKY85 (nrLSU), GTR+G (*rpb2* introns), GTR+G (*rpb2* exon), HKY85+I+G (*tef1-α* introns), GTR+G (*tef1-α* 1^st^ codon), HKY85+G (*tef1-α* 2^nd^ codon), and K80+G (*tef1-α* 3^rd^ codon). The combined dataset with partition-specific models was used for Bayesian analysis using MrBayes version 3.2.7a (Ronquist et al. 2012) with two independent runs comprising four Metropolis-coupled Markov chain Monte Carlo chains, each running for 4,000,000 generations with sampling every 1,000 generations. The first 25% of sampled trees were discarded as burn-in, and the remaining trees were used to infer a majority-rule consensus and calculate Bayesian posterior probabilities (BPP) for the clades. Maximum likelihood (ML) analyses were performed using raxmlGUI 2.0 (Edler et al. 2020), involving 1,000 replicates under the GTR+GAMMAI model. Additionally, 1,000 rapid bootstrap replicates were performed using the GTRCAT model to evaluate the ML bootstrap values of the nodes. A clade was considered strongly supported if bootstrap (BS) ≥ 60% and posterior probability (PP) ≥ 0.80. Newly generated sequences were deposited in GenBank (http://www.ncbi.nlm.nih.gov/) (Table 1). The alignment was submitted to Figshare (DOI: 10.6084/m9.figshare.29844794).

**Table 1.** Names, strain numbers, countries of collection, and corresponding GenBank accession numbers of the sequences used in this study. Newly generated sequences are in bold, ‘^T^’ indicates type specimens, and em dashes (—) indicate data unavailability.

Species	Strain	Country	ITS	LSU	tef1-α	rpb2
*Fuscoporia acutimarginata* ^T^	Dai 16892	China	MH050752	MH050766	MN848822	MH079393
*F. ambigua* ^T^	JV 0509/151	United States	MN816707	MN809996	—	MN848792
*F. americana* ^T^	BJFC 020644	United States	NR_173723	—	—	—
*F. atlantica*	VRTO24	Brazil	ON808610	ON795835	—	—
*F. australasica* ^T^	Dai 15636	China	MG008397	MG008450	MH636408	MH079402
*F. australiana* ^T^	Dai 18879	Australia	MN816705	MN810015	MN848850	MN848767
*F. bambusae* ^T^	Dai 16599	Thailand	MN816711	NG_075315	MN848808	—
*F. bambusicola* ^T^	Cui 8692	China	MN816739	MT032486	MN848813	—
*F. callimorpha*	Doll 868	China	MN816701	MN809992	MN848840	—
*F. caymanensis* ^T^	JV 1908/74	French Guiana	MT676832	MT676833	—	—
*F. centroamericana*	JV 1606/93	Costa Rica	MG008444	MG008460	MH636389	—
*F. chinensis* ^T^	Dai 15713	China	MN816721	MN810008	MN848846	MN848771
*F. chrysea*	JV 1607/106-J	Costa Rica	MN816736	MN810027	MN848818	MN848773
*F. cinchonensis*	CBS 447.76	South Korea	AY558613	—	—	—
*F. contigua* ^T^	Dai 16025	United States	MG008401	MG008454	MH636386	MH079406
*F. costaricana*	JV 1407/92	Costa Rica	MG008446	MG008461	—	—
*F. dhofarensis*	ATN-007	Oman	OP593104	OP593105	—	—
*F. dolichoseta* ^T^	SFC20191015-23	Republic of Korea	ON427765	ON427795	—	ON464731
*F. dollingeri* ^T^	Doll623	United States	MW908540	MW898444	—	—
*F. eucalypti ^T^*	Dai 18792	Australia	MN816731	MN810022	MN848831	MN848778
*F. eucalypticola* ^T^	Dai 18593A	Australia	PP732562	PP732631	—	—
*F. ferrea*	JV 1606/2.2-J	United States	KX961100	KY189100	MH636402	MH079394
*F. ferruginosa*	Cui 9244	China	MN816706	MN809995	MN848804	MN848791
*F. formasona* ^T^	VRTOBFO3	Brazil	ON808603	ON795827	—	—
*F. formasona*	VRTO83	Brazil	—	ON795830	—	—
*F. gilva*	JV 1209/65	United States	MN816719	MN810006	MN848851	MN848768
*F. gilvoides ^T^*	SFC20180426-12	Republic of Korea	ON427763	ON427793	ON479802	ON464729
*F. hawaiiana*	JV 2208/H22-J	United States	OQ817709	OQ817855	OQ849746	—
***F. indica* ^T^**	**MUBL1104**	**India**	—	**PP390498**	**PV638743**	**PV638735**
***F. indica***	**HRS-15B**	**India**	—	**PQ113747**	**PV638744**	**PV638736**
*F. insolita* ^T^	JV1208/5208	Russia	MN816724	MN810016	MN848800	—
*F. karsteniana* ^T^	Dai 11403	China	MN816717	MN810003	MN848795	MN848807
*F. koreana* ^T^	SFC20160726-93	Republic of Korea	ON427762	ON427792	ON479801	ON464728
*F. latispora*	JV 1109/482	United States	MG008439	MG008468	MH636395	MN848799
*F. licnoides*	URM 83001	Brazil	MH392561	MH407357	—	—
*F. minutissima* ^T^	JV 2208/H16-J	United States	OQ817711	OQ817857	OQ849748	—
*F. montana*	175856	Taiwan	JX484015	JX484007	—	—
*F. monticola* ^T^	Dai 11860	China	MG008406	MG008457	MH636390	—
*F. marquesiana*	URM83094	Brazil	MH392544	MH407343	—	—
*F. nadiatariana*	MUBL1105	India	PQ098039	PP390499	PQ346367	PQ346369
*F. nadiatariana*	SP2F2A	India	PQ098040	PQ113748	PQ346368	PQ346370
*F. palomari*	JV 1305/3-J	United States	MN816738	MN810028	—	—
*F. plumeriae* ^T^	Dai 18858	Australia	MN816712	MN810010	MN848843	MN848769
*F. pulviniformis* ^T^	CMW48600	South Africa	MH599102	MH599127	MT108960	—
*F. punctatiformis*	Dai 17443	China	MH050755	MH050764	—	—
*F. ramulicola* ^T^	Dai 15723	China	MH050749	MH050762	MN848824	MH079398
*F. resupinata*	Dai 20455	China	PP732567	PP732636	—	—
*F. reticulata* ^T^	SFC20160115-16	Republic of Korea	ON427761	ON427791	ON479800	ON464727
*F. rhabarbarina*	Dai 16226	China	MN816743	MN810035	MN848838	MN848784
*F. roseocinerea*	JV 1407/84	China	MN816740	MN810030	MN848819	—
*F. rufitincta*	JV 1008/25	United States	KJ940029	KX058575	—	—
*F. sarcites*	JV 0402/20K	Venezuela	MZ264225	MZ264218	—	—
*F. semiarida*	URM 82510	Brazil	MH392563	MH407362	—	—
*F. scruposa*	VRTOV473	Brazil		ON795836	—	—
*F. senex*	Dai 15775	China	MN816746	MN810038	—	—
*F. septiseta* ^T^	Dai 12820	United States	MG008405	MN810033	MH636394	—
*F. setifera*	Dai 15706	China	MH050759	MH050769	MN848842	MN159391
*F. shoreae* ^T^	Dai 17818	Singapore	MN816735	MN810026	MN848816	—
*F. sinica* ^T^	Dai 15468	China	MG008412	MG008459	MH636392	—
*F. sinuosa*	Dai 20499	China	MZ264227	MZ264220	—	—
***F. sirumalaiensis* ^T^**	**MUBL1106**	**India**	**PQ098037**	**PP390500**	**PV638741**	**PV638737**
***F. sirumalaiensis***	**SRM21**	**India**	**PQ098038**	**PQ113746**	**PV638742**	**PV638738**
*F. subchrysea* ^T^	Dai 16201	China	MN816708	MN809997	MN848811	MN848796
*F. subferrea* ^T^	Dai 16327	China	KX961098	KY053473	—	MH079401
*F. submurina* ^T^	Dai 19655	China	MZ264228	MZ264221	—	—
*F. subtropica* ^T^	Dai 19957	China	PP732565	PP732634	—	—
***F. terminalianae* ^T^**	**MUBL1107**	**India**	**PQ098033**	**PP390501**	**PV638739**	**PV638733**
***F. terminalianae***	**VM2B**	**India**	**PQ098034**	**PQ113745**	**PV638740**	**PV638734**
*F. torulosa*	JV 1405/2	Czech Republic	KX961106	KY189106	MH636405	MN159392
*F. viticola*	He 2081	United States	MN121829	MN121770	—	—
*F. wahlbergii*	JV 1312/20-Kout	Spain	MN81672	MG008462	—	—
*F. yunnanensis*	Cui 8182	China	MH050756	MN810029	—	MN848789
**Outgroup**						
*Coniferiporia weirii*	CFS 504	Canada	AY829341	AY829345	—	—
*Phellinidium fragrans*	CBS 202.90	Canada	AY558619	AY059027	—	—

## ﻿Results

### ﻿Molecular phylogeny

**Figure 1.**
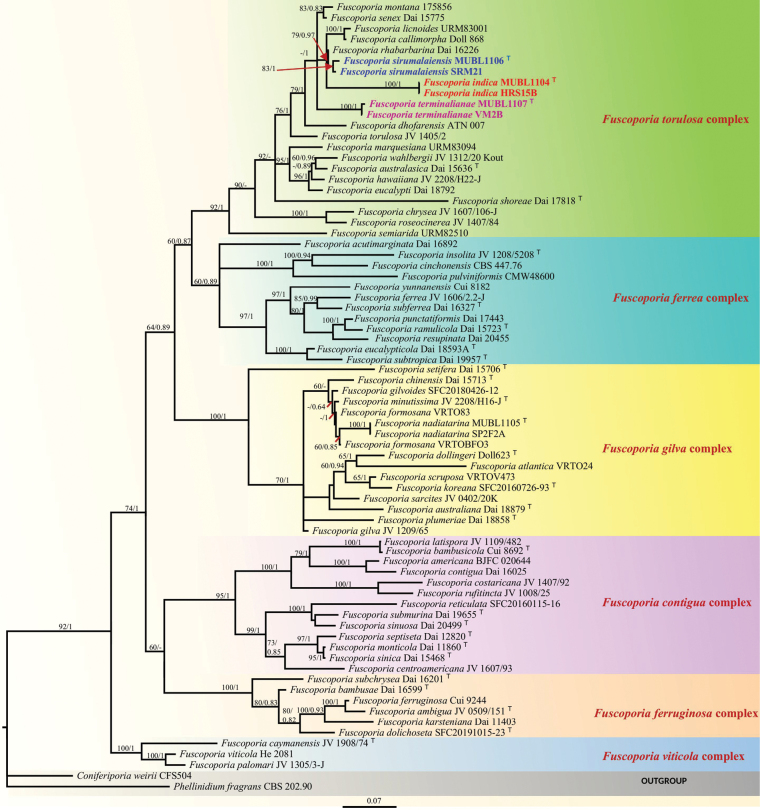
Phylogram generated from maximum likelihood analysis based on combined ITS, nrLSU, *rpb2*, and *tef1-α* sequence data of species of *Fuscoporia* with the outgroups *Phellinidium fragrans* (CBS 202.90) and *Coniferiporia weirii* (CFS504). Bootstrap support values ≥ 60% for ML and Bayesian posterior probabilities ≥ 0.80 are indicated above the nodes. Species complexes are highlighted, and newly generated sequences are shown in bold red, blue, and pink; type specimens are tagged with “^T.^”

In total, 22 new sequences of ITS, nrLSU, *rpb2*, and *tef1-α* generated in this study were submitted to GenBank, and the accession numbers are listed in Table 1. In addition, ITS, nrLSU, *rpb2*, and *tef1-α* sequences of 74 allied taxa (65 ITS, 72 nrLSU, 28 *rpb2*, and 39 *tef1-α*) were retrieved from GenBank (Table 1), along with the outgroups *Coniferiporia weirii* (CFS504) and *Phellinidium fragrans* (CBS 202.90). The concatenated multiple sequence alignment was 3,431 bases long, of which 1,854 were constant, 1,468 were variable, and 1,130 (33%) were parsimony informative. The ITS region comprised 879 bases, nrLSU 1,385 bases, *rpb2* 620 bases, and *tef1-α* 544 bases. Maximum likelihood (ML) and Bayesian inference (BI) analyses generated nearly identical tree topologies, with little variation in statistical support. Therefore, only the ML tree is shown (Fig. 1).

The phylogenetic analyses revealed six major clades representing six species complexes in *Fuscoporia*, which were consistent with earlier reports (Fig. 1) (Chen et al. 2020, 2023a, b). Our Indian specimens were recovered in the *F. torulosa* complex, which received robust phylogenetic support in both ML and BI analyses (92 ML/1.00 BI). *Fuscoporia sirumalaiensis* formed a sister clade to *F. rhabarbarina*; *F. indica* formed a sister taxon to *F. sirumalaiensis*, *F. rhabarbarina*, *F. licnoides*, and *F. callimorpha*, while *F. terminalianae* formed a distinct lineage within the *F. torulosa* complex. The newly identified species are characterized by effused-reflexed to pileate basidiocarps, smaller pores, septate skeletal hyphae, the presence of hymenial setae, the absence of mycelial setae, the presence of cystidioles, and broadly ellipsoid to subglobose basidiospores.

## ﻿Taxonomy

### ﻿ Fuscoporia indica

Taxon classificationFungiHymenochaetalesHymenochaetaceae

M. Kaliyaperumal, S. Gunaseelan & K. Kezo sp. nov.

666E4A48-EAC3-5CEE-958C-DD6DF86C6AC1

MB 859271

Fig. 2

#### Diagnosis.

*Fuscoporia indica* is diagnosed by its annual, applanate, dimidiate basidiome with smooth to glabrous, indistinctly zonate pilear surface, homogenous context, the presence of cystidioles, and ellipsoidal, acyanophilic basidiospores.

#### Holotype.

**India** • Tamil Nadu, Salem District, Yercaud, on hardwood, 23 January 2018, S. Gunaseelan, HRS-15A (**holotype** MUBL1104). GenBank: PP390498 (nrLSU); PV638735 (*rpb2*); PV638743 (*tef1-α*).

#### Etymology.

Refers to the type locality, “India.”

#### Description.

Basidiomes annual, pileate, applanate, sessile to imbricate, soft, to light corky when dry. Pilei dimidiate, convex, projecting up to 4.5 cm long, 7 cm broad, and 1 cm thick at the base. Pileal surface yellowish brown (5D8), brown (6E6) to dark brown (6F8), smooth to glabrous, indistinctly zonate. Margin brown (6E5), acute, margin sterile, light brown (6D8), 1 mm in thickness. Pore surface light brown (6D8) to dark brown (6F8). Pores round to angular, 6–8 per mm. Marginal setae absent. Context light brown (6D8), homogenous, up to 3 mm in thickness. Tube layer yellowish-brown (6D8) to brownish-yellow (5C7), hard and corky, with tubes up to 2 mm long.

***Hyphal system*.** Hyphal system dimitic; generative hyphae simple septate; tissue darkening but otherwise unchanged in KOH.

***Context*.** Generative hyphae, hyaline to pale yellow, thin to slightly thick-walled, branched, frequently septate, 2–4 µm; skeletal hyphae dominant, rust-brown, thick-walled with a medium to wide lumen, unbranched, rarely septate, more or less straight, and regularly arranged, 2.2–3.5 µm.

***Tubes*.** Generative hyphae, dominant at the dissepiment edges and subhymenium, thin-walled, frequently branched and septate, hyaline to pale yellow, 1.8–3.4 µm, some encrusted at dissepiment edges and in hymenium; skeletal hyphae dominant, thick-walled with a medium to wide lumen, more or less straight, subparallel along the tubes, yellow to golden yellow, 2–3.2 µm. Hymenial setae subulate to ventricose, acute to acuminate at the apex, encrusted, mostly originating from tramal hyphae, dark brown, thick-walled, 8−39 × 5−8 µm; Cystidioles hyaline to pale yellow in water, fusoid to subulate, rare, tapering at the end, 6–42 × 2.8–7 µm. Basidioles clavate to broadly clavate 6.5–18 × 2.5–6.5 µm. Basidia broadly clavate, hyaline, four sterigmata, 7–17 × 2.8–6.7 µm. Basidiospores ellipsoid, hyaline, thin-walled, smooth, CB^−^, IKI^−^, (3.2–)3.5–4.5(–4.8) × (2.2–)2.5–2.9(–3.2) µm, Q=1.5, Q range = 1.3–1.7, (n = 30/2).

**Figure 2.**
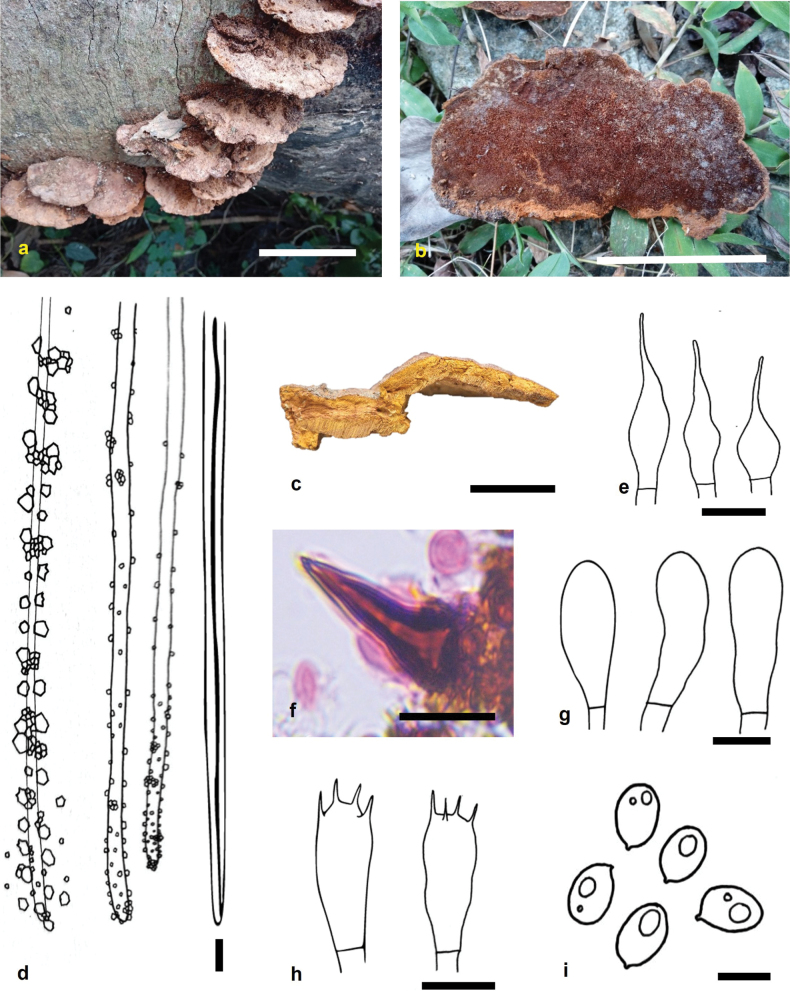
*Fuscoporia indica* (holotype MUBL1104). **a.** Basidiomes; **b.** Pore surface; **c.** Transverse section of a basidiome; **d.** Dissepiment edges and skeletal hyphae; **e.** Cystidioles; **f.** Hymenial setae; **g.** Basidioles; **h.** Basidia; **i.** Basidiospores. Scale bars: 5 cm (**a, b**); 2 cm (**c**); 5 μm (**d–i**).

#### Distribution.

India (Tamil Nadu).

#### Additional specimen examined (paratype).

**India** • Tamil Nadu, Salem District, Yercaud (11°79'63"N, 78°21'20"E), on hardwood, 23 January 2018, S. Gunaseelan, HRS-15B. GenBank: PQ113747 (nrLSU); PV638736 (*rpb2*); PV638744 (*tef1-α*).

#### Notes.

The phenetic characters of *F. indica* and *F. licnoides* are similar, both having a concentrically zonate, glabrous pileal surface, homogeneous context, and acute margin. However, *F. indica* differs in having imbricate, dimidiate, convex pilei and smaller basidiospores (*F. indica* 3.2–4.8 × 2.2–3.2 μm vs. *F. licnoides* 4–5 × 2.5–3.5 μm) (Oliveira and Gibertoni 2023). *Fuscoporia indica* differs from *F. callimorpha* in having an imbricate basidiome, convex pilei, smaller pores, and larger hymenial setae (Dai 2010). *Fuscoporia indica*, *F. senex*, and *F. torulosa* share similar features such as applanate, dimidiate basidiomes with a dimitic hyphal system, the presence of cystidioles, and cyanophilic basidiospores, but *F. indica* differs in having a glabrous, azonate pilear surface and larger basidiospores. *Fuscoporia indica* also differs from *F. rhabarbarina* by the absence of a crust above the context at maturity and, microscopically, by larger basidiospores. In contrast, *F. rhabarbarina* is identified by the presence of a crust above the context in older specimens and smaller spores (3.2–4.2 × 2–2.5 μm) (Dai 2010).

### ﻿ Fuscoporia sirumalaiensis

Taxon classificationFungiHymenochaetalesHymenochaetaceae

E. Arumugam, S. Gunaseelan & M. Kaliyaperumal sp. nov.

CFCC5B7B-A5D2-56B4-ADDF-210A983564FD

MB 859272

Fig. 3

#### Diagnosis.

Morphologically, *Fuscoporia sirumalaiensis* is unique with a perennial, imbricate to pileate, dimidiate basidiome, smooth to glabrous, concentrically zonate pilear surface, the presence of cystidioles, hymenial setae, and ellipsoidal basidiospores.

#### Holotype.

**India** • Tamil Nadu, Dindigul District, Sirumalai (10°28'23"N, 78°01'54"E), on hardwood, 31 Dec. 2022, M. Kaliyaperumal, SRM09 (holotype MUBL1106). GenBank: PP390500 (nrLSU); PQ098037 (ITS); PV638737 (*rpb2*); PV638741 (*tef1-α*).

#### Etymology.

Refers to the type locality “Sirumalai,” India.

#### Description.

Basidiomes biennial to perennial, imbricate to pileate, sessile, applanate. Pilei dimidiate, projecting up to 5.2 cm long, 7 cm wide, and 2 cm thick near the attachment. Pilear surface dark brown (6F7) to greyish brown (6E3), smooth to glabrous, concentrically zonate. Margin brown (6E8), acute, up to 1 mm. Context light brown (6D8) to brown (6E8), duplex, 0.4 cm. Pore surface, light brown (6D6) to brown (6E7). Pores circular, 5–8 per mm. Tubes light brown (6D8) to brown (6E8), up to 1 cm long, each stratum up to 0.2 cm.

***Hyphal system*.** Hyphal system dimitic; generative hyphae simple septate; tissue darkening but otherwise unchanged in KOH.

***Context*.** Generative hyphae rare, hyaline, thin to slightly thick-walled, branched, frequently septate, 2–5 µm; skeletal hyphae rust-brown, thick-walled with a narrow to wide lumen, unbranched, aseptate, 2.5–5 µm.

***Trama*.** Generative hyphae hyaline, thin to thick-walled, mostly present at dissepiment edges and subhymenium, frequently branched with simple septate, a few are encrusted at dissepiment edges and in the hymenium, 2.5–5.2 µm; skeletal hyphae dominant, yellowish brown, thick-walled with a medium to wide lumen, aseptate, subparallel along the tubes, 2.5–5.6 µm. Hymenial setae subulate to ventricose, indistinctly encrusted, dark brown, 20−45 × 5.2−9 µm. Cystidioles hyaline, fusoid to subulate, infrequent, tapering at the end, 9–28 × 2.5–7.2 µm. Basidioles broadly clavate 6.5–18 × 3.5–6.2 µm. Basidia broadly clavate, hyaline, four sterigmata, 6–17 × 4–6.5 µm. Basidiospores ellipsoid, hyaline, thin-walled, smooth, CB ¯, IKI ¯, (2.8–)3.1–3.6(–3.8) × (1.8–)2.1–2.6(–2.8) µm, Q=1.5, Q range = 1.2–1.6, (n = 30/2).

**Figure 3.**
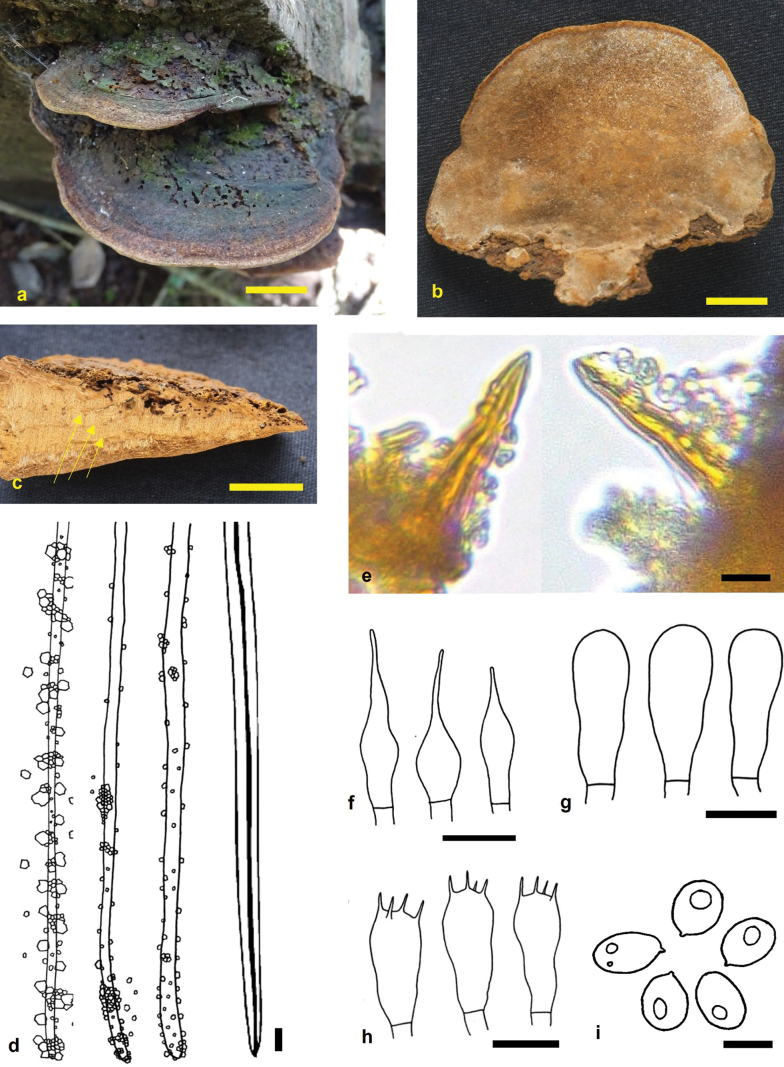
*Fuscoporia sirumalaiensis* (holotype MUBL1106). **a.** Basidiomes; **b.** Pore surface; **c.** Transverse section of a basidiome (arrows indicate the stratified tube layers); **d.** Dissepiment edges and skeletal hyphae; **e.** Hymenial setae; **f.** Cystidioles; **g.** Basidioles; **h.** Basidia; **i.** Basidiospores. Scale bars: 2 cm (**a–c**); 5 μm (**d–i**).

#### Distribution.

India (Tamil Nadu).

#### Additional specimen examined.

**India** • Tamil Nadu, Dindigul District, Sirumalai (10°27'73"N, 78°01'57"E), on hardwood, 31 December 2022, M. Kaliyaperumal, SRM21. GenBank: PQ113746 (nrLSU); PQ098038 (ITS); PV638738 (*rpb2*); PV638742 (*tef1-α*).

#### Notes.

Multigene phylogenetic analyses revealed that *Fuscoporia sirumalaiensis* forms a sister clade to *F. rhabarbarina* (83% ML/1 BI) within the *F. torulosa* complex (Chen et al. 2023a, b). *Fuscoporia sirumalaiensis* and *F. rhabarbarina* are similar only in their concentrically zonate brown basidiomes, but the two species differ greatly in other morphological characteristics such as the size of cystidioles, hymenial setae, and basidiospores (Dai 2010). The phenetic features of *F. sirumalaiensis* vary significantly from those of *F. callimorpha*, *F. dhofarensis*, and *F. licnoides* (Dai 2010; Oliveira and Gibertoni 2023). *Fuscoporia sirumalaiensis* differs from *F. indica* in having larger hymenial setae, differences in cystidiole size, and smaller basidiospores (*F. sirumalaiensis* (2.8–)3.1–3.6(–3.8) × (1.8–)2.1–2.6(–2.8) μm vs. *F. indica* (3.2–)3.5–4.5(–4.8) × (2.2–)2.5–2.9(–3.2) μm) (present study).

### ﻿ Fuscoporia terminalianae

Taxon classificationFungiHymenochaetalesHymenochaetaceae

E. Arumugam, S. Gunaseelan & M. Kaliyaperumal sp. nov.

4E9D40F2-3415-5E87-BB22-D1F6B92034B9

MB 859273

Fig. 4

#### Diagnosis.

Morphologically, *F. terminalianae* can be diagnosed by an annual, resupinate to effused, often pileate basidiome, infrequently warted, and widely zonate pilear surface with homogeneous context. Microscopically, the presence of cystidioles and ellipsoidal, acyanophilic basidiospores are characteristic features of this species.

#### Holotype.

**India** • Tamil Nadu, Kallakurichi District, Vellimalai (11°86'16"N, 78°70'16"E), on dead wood (*Terminalia bellerica*), 30 Oct. 2019, M. Kaliyaperumal, VM2A (**holotype** MUBL1107). GenBank: PP390501 (nrLSU); PQ098033 (ITS); PV638733 (*rpb2*); PV638739 (*tef1-α*).

#### Etymology.

Refers to the host tree *Terminalia bellerica* on which the fungus was collected.

#### Description.

Basidiome annual, resupinate to effused, reflexed, pileate, applanate, sessile, fused to imbricate, soft when fresh, corky when dry. Pilei dimidiate, projecting up to 2.8 cm long, 5.4 cm wide, and 3.2 cm thick at the base. Pileal surface light brown (6D5), brown (6E8) to dark brown (6F8), smooth, widely zonate, infrequently warted. Margin brown (6E8), velutinate, obtuse to acute, up to 3 mm in thickness. Marginal setae absent. Pore surface brown (6E7) to dark brown (6F8), glancing. Pores round to angular, 6–9 per mm. Context light brown (6D8), zonate, homogenous, up to 3 mm in thickness. Tube layer golden brown (5D7) to light brown (6D8), hard and corky, with tubes up to 2 mm long.

**Figure 4.**
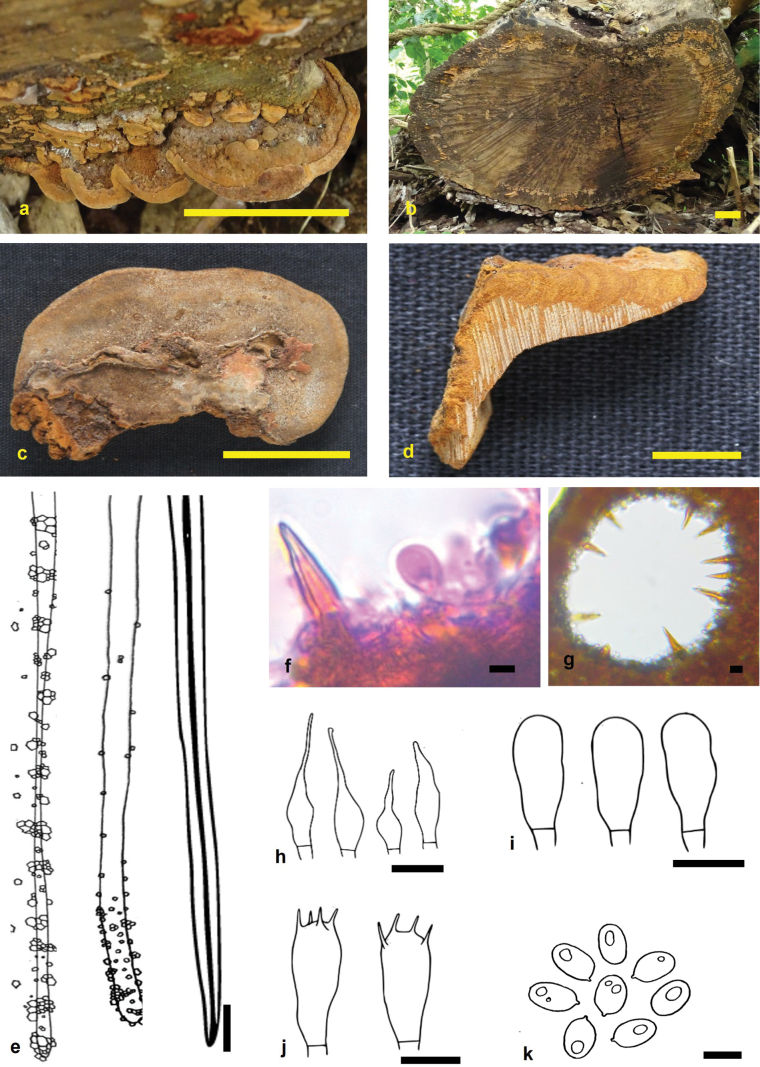
*Fuscoporia terminalianae* (holotype MUBL1107). **a.** Basidiomes; **b.** Habitat; **c.** Pore surface; **d.** Transverse section of a basidiome; **e.** Dissepiment edges and skeletal hyphae; **f, g.** Hymenial setae; **h.** Cystidioles; **i.** Basidioles; **j.** Basidia; **k.** Basidiospores. Scale bars: 2 cm (**a–d**); 5 μm (**e–k**).

***Hyphal system*.** Hyphal system dimitic; generative hyphae simple septate; tissue darkening but otherwise unchanged in KOH.

***Context*.** Generative hyphae hyaline to golden yellow, thin to thick-walled, branched, frequently septate, 2–5 µm; skeletal hyphae dominant, rust-brown, thick-walled with a medium to wide lumen, unbranched, occasionally septate, 2.5–4.8 µm.

***Tubes*.** Generative hyphae mostly present at dissepiment edges and subhymenium, hyaline to pale yellow, thin to thick-walled, frequently branched and simple septate, 1.5–5.2 mm, most of the hyphae at dissepiment edges and hymenium are encrusted with simple crystals, skeletal hyphae dominant, yellowish brown, thick-walled with a medium to wide lumen, frequently septate, more or less straight, subparallel along the tubes, 2.5–5 mm. Hymenial setae ventricose to subulate, mostly originating from tramal hyphae, dark brown, thick-walled, 13−45 × 4−7 µm. Cystidioles hyaline, fusoid to subulate, rare, tapering at the end, 9–32 × 4.5–7 µm. Basidioles broadly clavate 5–13 × 2.5–6 µm. Basidia broadly clavate, hyaline, four sterigmata, 5.2–15 × 2.9–6 µm. Basidiospores ellipsoid, hyaline, thin-walled, smooth, CB ¯, IKI ¯, (3–)3.3–4.3(–4.5) × (2.5–)2.8–3 µm, Q=1.48, Q range=1.2–1.7, (n = 30/2).

#### Additional specimen examined.

**India** • Tamil Nadu, Kallakurichi District, Vellimalai (11°87'77"N, 78°69'52"E), on dead wood (*Terminalia bellerica*), 30 Oct. 2019, M. Kaliyaperumal, VM2B. GenBank: PQ113745 (nrLSU); PQ098034 (ITS); PV638734 (*rpb2*); PV638740 (*tef1-α*).

#### Notes.

In multigene phylogenetic analyses, *F. terminalianae* forms an isolated lineage within the *F. torulosa* complex. *Fuscoporia rhabarbarina* is distinguished from *F. terminalianae* by its glabrous, sulcate pilear surface, number of pores, and basidiospore size; in contrast, *F. terminalianae* has a smooth, infrequently warted, widely zonate pilear surface (Dai 2010). *Fuscoporia licnoides* and *F. terminalianae* are similar in having a zonate basidiome and homogeneous context, but the latter differs in having an effused-reflexed basidiome, larger setae, and smaller basidiospores (*F. terminalianae* 3–4.5 × 2.5–3 μm vs. *F. licnoides* 4–5 × 2.5–3.5 μm) (Oliveira and Gibertoni 2023). *Fuscoporia terminalianae*, *F. senex*, and *F. torulosa* are similar in having applanate, dimidiate basidiomes with narrowly zonate pilear surfaces and dimitic hyphal systems, as well as the presence of cystidioles and cyanophilic basidiospores, while *F. terminalianae* differs in having a warted pilear surface and smaller basidiospores (Dai 2010).

## ﻿Discussion

The present study contributes to the taxonomy of Indian representatives of the genus *Fuscoporia* by describing new taxa using an integrative approach that combines morphological observations with multimarker phylogenetic analyses. Compared with information available from classical taxonomy, phylogenetic analyses have been significant in resolving species limits and clarifying evolutionary relationships, enabling a more comprehensive classification of the taxa.

Chen et al. (2020) used phylogenetic analysis to reveal six major complexes within *Fuscoporia*. These were (i) the *F. contigua* group (resupinate basidiome, moderately large pores, the presence of mycelial setae and hymenial setae, and ellipsoid to broadly ellipsoid basidiospores), (ii) the *F. ferrea* group (resupinate basidiome, aseptate skeletal hyphae, absence of mycelial setae, the presence of hymenial setae and cystidioles, and cylindric basidiospores), (iii) the *F. ferruginosa* group (resupinate basidiome, relatively small pores, entire dissepiments, straight hymenial setae, the presence of mycelial setae, and ellipsoid basidiospores), (iv) the *F. gilva* group (effused-reflexed to pileate basidiome, indistinctly concentrically sulcate zones, hispid to rugose or nodulose pilear surface, lacerate dissepiments, the presence of cystidioles, and ellipsoid to cylindric basidiospores), (v) the *F. torulosa* group (resupinate, effused-reflexed to pileate basidiome, smaller pores, septate skeletal hyphae, straight or hooked hymenial setae, absence of mycelial setae, the presence of cystidioles, and broadly ellipsoid to subglobose basidiospores), and (vi) the *F. viticola* group (resupinate to effused-reflexed basidiome, moderately large pores, absence of mycelial setae, narrowly subulate and long hymenial setae, and cylindric, long basidiospores) (Chen et al. 2019, 2020).

Over the past decade, there has been significant progress in understanding the diversity and geographical distribution of *Fuscoporia* species (Dai 2010; Raymundo et al. 2013; Chen et al. 2019, 2020, 2023a, b; Hussain et al. 2022; Oliveira and Gibertoni 2023; Crous et al. 2024). India is characterized by rich vegetation and several biodiversity hotspots; nevertheless, limited attention has been devoted to the diversity of hymenochaetoid fungi. The few conventional taxonomic studies undertaken were confined to the northern parts of the country (Singh et al. 2014; Kumari et al. 2021). As described herein, we sampled and delimited three new species of *Fuscoporia* based on morpho-taxonomic characters and phylogenetic relationships ascertained from a concatenated ITS+nrLSU+*rpb2*+*tef1-α* sequence dataset. The three species—*F. indica*, *F. sirumalaiensis*, and *F. terminalianae*—were recovered in the *F. torulosa* complex (92% ML/1 BPP) (Fig. 1). They show significant variation in morpho-microscopic characters, and the grouping is consistent with the literature (Chen et al. 2020, 2023a, b). Recently, our team reported *F. naditirana* from the Eastern Ghats of India, a species recovered within the *F. gilva* complex (Crous et al. 2024).

Phylogenetically, the newly described species were recovered within the *F. torulosa* complex, which accommodates species characterized by resupinate, effused-reflexed to pileate basidiomes with septate skeletal hyphae, the presence of cystidioles, straight or hooked hymenial setae, and broadly ellipsoid to subglobose basidiospores (Chen et al. 2020, 2023a, b). Morphologically, *F. terminalianae* differs from *F. indica* and *F. sirumalaiensis* by having resupinate to imbricate and/or pileate basidiomes with infrequently warted pilear surfaces, obtuse margins, and larger basidiospores (3–4.5 × 2.5–3 μm). Likewise, *F. sirumalaiensis* differs from *F. indica* by its basidiome with a smooth to glabrous, narrowly zonate pilear surface, duplex context, larger pores (5–8/mm), and smaller basidiospores (2.8–3.8 × 1.8–2.8 μm).

Nevertheless, *F. indica*, *F. sirumalaiensis*, and *F. terminalianae* are phylogenetically and morphologically distinct from close allies and other species in the *F. torulosa* complex—*F. australasica*, *F. callimorpha*, *F. chrysea*, *F. dhofarensis*, *F. eucalypti*, *F. hawaiiana*, *F. licnoides*, *F. montana*, *F. marquesiana*, *F. rhabarbarina*, *F. senex*, *F. roseocinerea*, *F. shoreae*, *F. torulosa*, and *F. wahlbergii* (Chen et al. 2020, 2023a, b). The Indian species of *Fuscoporia* described herein have pileate basidiomes and lack hyphidia, whereas *F. montana* is characterized by completely resupinate basidiomes, occasionally with swollen edges, and the presence of hyphidia (Dai 2010; Chen et al. 2020). Likewise, *F. chrysea* differs from the three Indian species of *Fuscoporia* in having resupinate to effused-reflexed basidiomes that rarely form a narrow pileus or pseudo-pileus (Raymundo et al. 2013). Despite sharing pileate, applanate to dimidiate basidiomes, *F. indica*, *F. sirumalaiensis*, and *F. terminalianae* differ from *F. australasica*, *F. eucalypti*, *F. marquesiana*, and *F. wahlbergii* in having straight hymenial setae with acute tips, whereas the latter species are reported to have hooked hymenial setae (Dai 2010; Chen et al. 2020; Yuan et al. 2020). The absence of setae makes *F. shoreae* distinct from the Indian *Fuscoporia* species (Chen et al. 2020).

## ﻿Conclusion

Considering the significance of *Fuscoporia* in pharmacognosy and ecology, as well as the taxonomic disarray within the Hymenochaetaceae, effective species identification is crucial for future research. Taxonomic classification within the genus has remained challenging because of overlapping morphological traits and the limited resolution of the routinely used ITS region. In India, species of *Fuscoporia* were historically misclassified as *Phellinus* based solely on morphological characteristics. Our study highlights the importance of employing multimarker phylogenetic analysis in conjunction with detailed morphological observations to delineate the three novel species within the *F. torulosa* complex collected from the Eastern Ghats of Tamil Nadu. Furthermore, additional sampling from the understudied regions of Tamil Nadu, particularly the Western Ghats, is likely to enhance our understanding of the taxonomic complexity and species diversity of *Fuscoporia*.

## Supplementary Material

XML Treatment for Fuscoporia indica

XML Treatment for Fuscoporia sirumalaiensis

XML Treatment for Fuscoporia terminalianae

## ﻿Additional information

### Use of AI

No use of AI was reported.

### Author ORCIDs

Sugantha Gunaseelan https://orcid.org/0000-0001-7089-2292

Kezhocuyi Kezo https://orcid.org/0000-0002-3723-0462

Elangovan Arumugam https://orcid.org/0009-0003-9720-2978

Samantha C. Karunarathna https://orcid.org/0000-0001-7080-0781

Abdallah M. Elgorban https://orcid.org/0000-0003-3664-7853

Jaturong Kumla https://orcid.org/0000-0002-3673-6541

Nakarin Suwannarach https://orcid.org/0000-0002-2653-1913

Ekachai Chukeatirote https://orcid.org/0000-0002-9968-5841

Malarvizhi Kaliyaperumal https://orcid.org/0000-0002-1218-3778

### Data availability

All holotype and paratype collections of the new species are deposited at the Madras University Botany Laboratory (MUBL), Centre for Advanced Studies in Botany, University of Madras, Chennai 600025, Tamil Nadu, India. The sequences generated during this study are deposited in NCBI GenBank. The ITS, nrLSU, partial *rpb2*, and *tef1*-α alignment is deposited in FigShare.
